# Non-wood Fibre Production of Microcrystalline Cellulose from *Sorghum caudatum*: Characterisation and Tableting Properties

**DOI:** 10.4103/0250-474X.70473

**Published:** 2010

**Authors:** F. O. Ohwoavworhua, T. A. Adelakun

**Affiliations:** Department of Pharmaceutical Technology and Raw Materials Development (PT and RMD), National Institute for Pharmaceutical Research and Development, (NIPRD), P.M.B. 21, Abuja, Nigeria

**Keywords:** Characterization, extraction, microcrystalline cellulose, *Sorghum caudatum*, tableting

## Abstract

The microcrystalline cellulose is an important ingredient in pharmaceutical, food, cosmetic and other industries. In this study, the microcrystalline cellulose, obtained from the stalk of *Sorghum caudatum* was evaluated for its physical and tableting characteristics with a view to assessing its usefulness in pharmaceutical tableting. The microcrystalline cellulose, obtained from the stalk of *Sorghum caudatum*, obtained by sodium hydroxide delignification followed by sodium hypochlorite bleaching and acid hydrolysis was examined for its physicochemical and tableting properties in comparison with those of the well-known commercial microcrystalline cellulose grade, Avicel PH 101. The extraction yield of this microcrystalline cellulose, obtained from the stalk of *Sorghum caudatum* was approximately 19%. The cellulose material was composed of irregularly shaped fibrous cellulose particles and had a moisture content of 6.2% and total ash of 0.28%. The true density was 1.46. The flow indices showed that the microcrystalline cellulose, obtained from the stalk of *Sorghum caudatum* flowed poorly. The hydration, swelling and moisture sorption capacities were 3.9, 85 and 24%, respectively. Tablets resulting from these cellulose materials were found to be without surface defects, sufficiently hard and having disintegration time within 15 min. The study revealed that the microcrystalline cellulose, obtained from the stalk of *Sorghum caudatum* compares favourably with Avicel PH 101 and conformed to official requirement specified in the British Pharmacopoeia 1993 for microcrystalline cellulose.

Environment friendly or green products are those that use less environmental resources, emit less pollutants to the different environment, serve as substitute for more valuable resources, utilize waste for the production of valuable materials (resources recovery), and conserve energy in industrial processing[1,2]. The utilization of materials commonly viewed as waste, also designated resource recovery, is a new trend in sustainable development. Wastes are considered secondary material resources and at the same time are renewable material resources.

Microcrystalline cellulose (MCC) for industrial purposes is usually obtained from wood pulp and purified cotton linters. Each of these is a “natural” source, cotton is a high value-added crop and wood pulp generally originates in some manner from deforestation. The need for environment friendly processes as well as the need to slow down the fast global deforestation has stimulated renewed interest in agro-fiber plants waste[3]. It is against this background that the stalk from *Sorghum caudatum*, which occur as huge agricultural waste in Nigeria, was investigated as a source for the production of microcrystalline cellulose. Alternative sources for MCC recently investigated include agricultural wastes and other plants parts not traditionally used for MCC production[4–9].

*Sorghum caudatum*, commonly known as guinea corn, is an annual plant of the grass family Gramineae. It is cultivated most extensively for human food as a rainy season crop in the seasonally dry African and Asian savanna zones, especially in West Africa and India. In these areas it is often the staple food crop and as such, it often receives priority for land and labour over other crops. Besides, sorghum grain is used in peasant and subsistence farming communities to make beer and its strong stalk are valued for fencing and for the construction of temporary buildings. Its grain also finds use as livestock food[10]. Stalks of some species in the family of Gramineae like wheat (*Triticum spp*), guinea corn (*Sorghum bicolour*), and sugar cane (*Saccharum officinarum*), which exist as huge waste after the grains are harvested, have been reported in the literature as sources of α-cellulose and or its modified form, MCC[4,8,9]. This work reports on the preparation, characterisation and tableting properties of a grade of microcrystalline cellulose, coded SC-MCC, prepared from α-cellulose content of the stalks of *S. caudatum*. Its properties were compared with those of the well-known commercial grade microcrystalline cellulose: Avicel PH 101.

## MATERIALS AND METHODS

All chemicals used other than specifically noted were of analytical grade. Water was double distilled. Other materials include sodium hydroxide (BDH, England), sodium hypochlorite (Jik^®^ Reckitt and Colman Ltd, Nigeria), hydrochloric acid (Fisons, UK), Avicel PH 101 (FMC Corporation, USA), xylene, phloroglucinol and iodine crystals (Hopkin and Williams, London) were used as obtained. All other instruments used were optical microscope, Nikon model Larphot 2 (Nikon Inc. Japan), pH meter Corning, Model 10 (England), sieve shaker machine (Endicott’s Ltd UK), Stampfvolumeter Model STAV 2003 JEF (Germany), vortex mixer (Vortex-Genie Scientific Industry, USA), Gallenkamp bench centrifuge (Gallenkamp, England), hydraulic hand press tableting machine (Model C, Carver Inc, USA), Erweka GmbH hardness tester Model: HT (Germany), Erweka disintegration test apparatus ZT4 (Germany), Erweka double drum Friabilator tester (Copley TAR Erweka, Germany).

Matured stalks of sorghum, properly identified in the herbarium unit of National Institute for Pharmaceutical Research and Development, Abuja, Nigeria, were collected from a farm in the Institute’s surrounding. The microcrystalline cellulose (SC-MCC) was prepared in our laboratory as described below.

### Extraction of α-cellulose:

The stalks were cut into small pieces and then crushed in a large mortar to squeeze out the juice. The resulting fibres were washed several times with water and air dried for five days and pulverized using a mill powered by an electric motor (3.7 kW/220 V). A fraction of the powdered material passing through a sieve of 2.0 mm aperture was used for extraction of α-cellulose as described in an earlier study[7].

### Production of MCC:

The procedure reported earlier[7], with slight modification, was used. A 50 g quantity of the α-cellulose obtained was placed in a glass container and hydrolyzed with 0.8 l of 2.5 N hydrochloric acid at a boiling temperature of 105° for 15 min. The hot acid mixture was poured into cold tap water which was followed by vigorous stirring with a wooden spatula and allowed to stand overnight. The microcrystalline cellulose obtained by this process was washed with water until neutral, filtered, pressed and dried in a fluid bed dryer at an inlet air temperature range of 57-60° for 60 min. Following further milling and sieving, the fraction passing through 710 µm sieve was obtained and stored at room temperature in a desiccator.

### Physicochemical properties of SC-MCC:

The organoleptic characteristic, identification tests, solubility, and presence of organic impurities, starch, dextrin and water-soluble substances were carried out in accordance with BP specifications[11]. The Nikon optical microscope was used for preliminary assessment of the nature of particles in SC-MCC powder particles. The combination of low and high power objective lenses of ×100 and ×400 magnification, respectively, were used.

pH determination was carried out by shaking 2 g of the powder material with 100 ml of distilled water for 5 min. and the pH of the supernatant liquid determined using a Corning pH meter[7]. Total ash content was estimated by measuring the residue left after combustion in a furnace at 550°[7].

### Powder properties:

The extracted SC-MCC and Avicel powders were evaluated for particle size analysis[12], density and compressibility index[6,7], angle of repose[13], powder porosity[7], hydration[14] and swelling capacity[7], moisture sorption capacity[6] and moisture content[6].

Particle size analysis was determined using a sieve shaker, containing test sieves ranging from 1.18 mm to 75 µm aperture size were arranged in a descending order. A 20 g quantity of SC-MCC powder was placed on the top sieve and the set-up was shaken for 5 min. The weight of material retained on each sieve determined. The average diameter was calculated as reported by Ansel *et al*.[12] using the equation: Average diameter =

(1) $$\left[\Sigma \;\left(\%\;\mathrm{retained}\right)\times \left(\mathrm{mean}\;\mathrm{aperture}\right)\right]/100.$$.

The true density, D_t_ of cellulose powders were determined by the liquid displacement method using xylene as the immersion fluid[6] and computed according to the following Eqn.,

(2) $$D_{t}=\;w/\left[\left(a+w\right)-b\right]\times \mathrm{SG}$$,

where w represents weight of powder, SG represents specific gravity of solvent, a represents weight of bottle+solvent and b represents weight of bottle+solvent+powder.

The static angle of repose, *θ*, was measured according to the fixed funnel and free standing cone method[13]. A funnel was clamped with its tip 2 cm above a graph paper placed on a flat horizontal surface. The powders were carefully poured through the funnel until the apex of the cone thus formed just reached the tip of the funnel. The mean diameters of the base of the powder cones were determined and the tangent of the angle of repose calculated using the Eqn.,

(3) Tan θ = 2h/D,

where h is the height of the heap of powder and D is the diameter of the base of the heap of powder.

For bulk and tap density determination, a 10 g quantity each of the powder samples was placed into a 50 ml clean, dry measuring cylinder and the volume, V_o_, occupied by each of the samples without tapping was determined. After 500 taps using Stampfvolumeter, occupied volumes, V_500,_ were determined. The bulk and tap densities were calculated as the ratio of weight to volume (V_0_ and V_500_ respectively)[7]. The Hausner index, compressibility index (C%) and powder porosity were calculated using equations, Hausner index= (Tapped density/bulk density)[7]; compressibility index (C%)= (Tapped density–bulk density)/Tapped density×100 %[7]; powder porosity =1-(bulk density/true density)×100, respectively.

The method of Kornblum and Stoopak[14] was adopted in the determination of hydration capacity. One gram each of the samples was placed in each of four 15 ml plastic centrifuge tubes and 10 ml of distilled water added and stoppered. The contents were mixed on a Vortex-Gennie vortex mixer for 2 min. The mixture was allowed to stand for 10 min. and immediately centrifuged at 1000 rpm for 10 min. on a Gallenkamp bench centrifuge. The supernatant was carefully decanted and the sediment was weighed. The hydration capacity was taken as the ratio of the weight of the sediment to the dry sample weight. Swelling capacity was measured at the same time as the hydration capacity determination using the method reported earlier[7] and calculated as follows:

(4) $$S=\;\left(V_{2}–V_{1}\right)/V_{1}\times 100$$,

where S is the % swelling capacity, V_2_ is the volume of the hydrated or swollen material and V_1_ is the tapped volume of the material prior to hydration.

Moisture sorption capacity was determined using 2 g of the cellulose material, accurately weighed and evenly distributed over the surface of a 70 mm tarred Petri dish. The samples were then placed in a large desiccator containing distilled water in its reservoir (RH = 100%) at room temperature and the weight gained by the exposed samples at the end of a five-day period was noted. The amount of water sorbed was calculated from the weight difference[6]. Moisture content was measured using 5 g of powder samples transferred into a Petri dish and then dried in an oven at 60° until a constant weight was obtained. The % moisture content was then determined as the ratio of moisture loss (g) to weight of sample expressed as percentage[6].

### Preparation of tablets:

SC-MCC and Avicel PH 101 compacts, each weighing 300 mg, were produced by compressing the powder for 1 min. with predetermined load (at a fixed compression pressure of 1.2 ton equivalent of 136 MPa) using a hydraulic hand press. Before each compression the die (10.5 mm in diameter) and the flat-faced punches were lubricated with a 1% (m/V) dispersion of magnesium stearate in ethanol. A total of 50 tablets were produced per batch.

Dicalcium phosphate dihydrate (DCP) tablets of this filler were made containing SC-MCC and Avicel PH 101 as disintegrant, at fixed compression force as above. A general formula was used as shown in Table 1. One batch contained SC-MCC while the other was prepared using Avicel PH 101. The materials were mixed in a bottle for 5 min. in each batch after which the resulting blend was compressed.

**TABLE 1:** FORMULA FOR INCORPORATING CELLULOSE MATERIALS IN DCP

Material	Weight (mg)
Dicalcium phosphate dehydrate (DCP)	268.5
Cellulose material	30
Magnesium stearate	1.5
Total weight of tablet	300.0

### Evaluation of tablets:

Compressed tablets were then evaluated for tablet strength, Disintegration time and friabilty. Tablets strength was determined using Erweka GmbH hardness tester which measures the force required to break a tablet. The mean of ten readings was reported. For the disintegration time determination, six tablets were assessed in a BP (1993) disintegration test apparatus ZT4 (Erweka) with distilled water at a temperature of 37° as medium. The mean of two determinations was reported. The tablets were subjected to friabillation in an Erweka double drum friabilator tester at 25 rpm for 4 min. The mass of ten tablets before and after the test, was taken. The percent loss of mass was calculated to obtain friability. The mean of two such determinations was reported.

### Statistical analysis:

The data were analysed statistically using Excel 2000 Windows^™^ (Microsoft Corporation).

## RESULTS AND DISCUSSION

The yield of α-cellulose was about 23 % w/w of the original material. The yield of the microcrystalline SC-MCC, obtained from α-cellulose was approximately 83 % w/w. Thus the yield of SC-MCC was approximately 19 % w/w of the starting dry plant material. The results of the physicochemical properties investigated are shown in Table 2. The results indicate a high level of purity of the cellulose material. The organoleptic properties of the SC-MCC produced were good as the material was odourless, tasteless, white and granular in texture. The value obtained for the total ash was very low possibly because cellulosic materials are almost free of inorganic compounds. When vegetable plants are incinerated, they leave an inorganic ash which in the case of many drugs varies within wide limits. The total ash value is of importance and indicates to some extent the amount of care taken in the preparation of the substance[3].

**TABLE 2:** SOME PHYSICOCHEMICAL PROPERTIES OF SC-MCC

TESTS	SC-MCC
Organoleptic Identification	Odourless, white, tasteless, coarse powder Turns violet-blue with iodinated ZnCl_2_
Organic impurities	Nil
Starch and dextrins	Nil
pH	6.8
Solubility (in ammonical solution of copper tetra-amine)	Complete and no residue
Water-soluble substance	< 0.2%
Total ash (%)	0.14 (0.01)
Microscopy	Irregularly shaped fibrous particles which are mixture of primary particles and spherical aggregates

Values in parenthesis indicate standard deviation for three determinations. DCP is dicalcium phsphate dehydrate

**Fig. 1:**
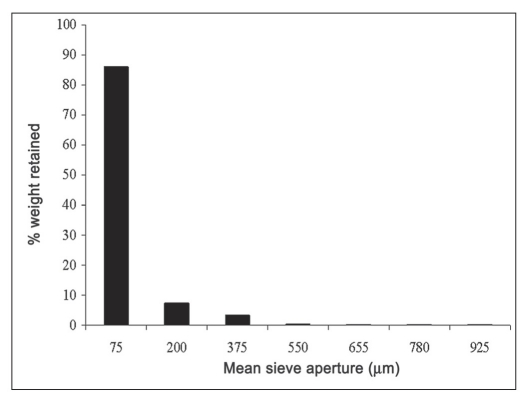
Particle size distribution of SC-MCC ■ SC-MCC is microcrystalline cellulose from *Sorghum caudatum*.

The powder properties of SC-MCC and Avicel PH 101 are presented in Table 3while the results of particle size analysis of SC-MCC powder is as shown in fig. 1. The figure represents a unimodal frequency distribution which is positively skewed. The particle size is in the range of 70-1000 µm, and as such, SC-MCC powder can be classified as a conventional powder[15]. Over 93 % of the particles population fell within 0-200 µm and the calculated average diameter was 95 µm.

**TABLE 3:** POWDER PROPERTIES OF SC-MCC AND AVICEL PH 101

Parameters	SC-MCC	Avicel PH 101
True density (g/ml)	1.46 (0.08)	1.40 (0.06)
Bulk density (g/ml)	0.27 (0.01)	0.31 (0.04)
Tapped density (g/ml)	0.48 (0.0)	0.42 (0.12)
Porosity (%)	81.60	78.00
Flow properties:		
(a) Angle of repose (°)	52.17(0.0)	41.20 (0.46)
(b) Hausner index	1.71	1.35
(c) Compressibility index (%)	43.75	26.00
Hydration capacity	3.90 (0.01)	2.17 (0.01)
Swelling capacity (%)	85.00 (3.5)	21.40 (0.03)
Moisture sorption capacity (%)	24.00 (1.6)	16.60 (0.24)
Moisture content (%)	6.20 (1.12)	7.40 (0.4)

Values in parenthesis indicate standard deviation for three determinations. SC-MCC is microcrystalline cellulose from *Sorghum caudatum*.

The true density of SC-MCC was comparable (*p*<0.05) to that of Avicel PH 101 (Table 3). Stamm[16] had pointed out that a direct correlation exists between the degree of crystallinity of cellulose and its true density when determined in a non-polar liquid. Consequently, the true density values for both cellulose materials suggest that they might have the same degree of crystallinity.

The moisture content of SC-MCC was about 5.2% which is well below the official limit of 8 % stated in British Pharmacopoeia, 1993[11]. This low value is indicative of the suitability of SC-MCC as a diluent in the formulation of hydrolysable drugs such as aspirin.

The flow properties of a powder are essential in determining the suitability of a material as a direct compression excipient. The angle of repose, Hausner index and Carr’s percent compressibility are considered as indirect measurements of powder flowability[17]. The high angle of reposes for both SC-MCC and Avicel PH 101 (Table 3) are indicative of poor flow[18], while the Hausner index is indicative of interparticle friction, and the Carr’s index shows the aptitude of a material to diminish in volume[17]. As the values of these indices increase, the flow of the powder decreases. In general however, Hausner ratio greater than 1.25 indicates poor flow and Carr’s compressibility index below 16 % indicates good flowability while values above 35 % indicate cohesivenes[17]. The flow indices showed that SC-MCC and Avicel PH 101 powders have poor flow. Consequently, a glidant will be needed when these materials are to be used in solid dosage production processes.

Swelling, which is generally accepted as an indication of tablet disintegration ability[19] can be assessed by the determination of hydration capacity, swelling capacity and moisture sorption profile. The hydration capacity value obtained for SC-MCC, (Table 3), indicates that it is capable of absorbing about four times its own weight of water. The swelling capacity, which reflects the increase in volume of cellulose following water sorption, was 85 % (Table 3). This is an indication that only a small portion of absorbed water actually penetrated the individual cellulose particles causing them to swell. The bulk of the absorbed water probably exists in a ‘free’ state between the particles. Thus, if the cellulose was incorporated in tablet formulation as a disintegrant it would probably produce tablet disintegration by two mechanisms: capillary or wicking due to interparticulate water, and swelling. In addition, the higher hydration and swelling capacities values observed for SC-MCC irrespective of comparable (*p*<0.05) powder porosity values of SC-MCC and Avicel PH101 (Table 3) could possibly be due to the difference in the proportion of amorphous cellulose present in the cellulose powders. Stamm[16] has reported that the amorphous portion is responsible for uptake and swelling of cellulose materials.

The moisture sorption capacity is a measure of moisture sensitivity of material. The moisture capacity value for SC-MCC was significantly different (*p*<0.05) from that of Avicel PH 101 value (Table 3). It has been reported that the crystallite portion of cellulose does not adsorb water and that the extent of water adsorption by cellulose should thus be proportional to the amount of amorphous cellulose present[16]. Thus, the result is indicative of the higher amount of amorphous cellulose likely present in SC-MCC unit fibrils. Also, study of water sorption is of importance since it reflects the relative physical stability of tablets when stored under humid conditions. Basically, this property showed that the cellulose powders are sensitive to atmospheric moisture and should therefore be stored in air tight container.

The tablets formed were good and acceptable as none had surface defects. Table 4 showed that the microcrystalline cellulose powders (SC-MCC and Avicel PH 101) have binding and disintegrant properties. The crushing strength values of tablets from SC-MCC and Avicel PH 101, at the compression force used, were comparable. Tablet crushing strength gives an indication of the ability of tablets to resist pressure. Crushing strength measurements can also be a useful tool in the preliminary screening of potential direct compression excipient[20]. Friability is related to the strength of the tablets and its ability to withstand abuse during normal handling, packaging and shipping[20]. All compacts had friability values less than 1 %. The friability and crushing strength values clearly demonstrate the high compactibility of the cellulose powders tested. The disintegration time of tablets of both materials fell within the 15 min. BP 1993 limits for conventional tablets. It should be observed however that despite the high swelling capacity of SC-MCC powder (Table 3), its disintegration time is longer. This could be attributed to its overwhelming adhesive properties.

**TABLE 4:** TABLETING PROPERTIES OF TWO MICROCRYSTALLINE CELLULOSE POWDERS

Direct compression filler-binder	Crushing strength (kgf)	Friability	Disintegration time (min)
SC-MCC	23.6 (2.90)	0.0	14.4 (1.40)
Avicel PH 101	22.4 (1.20)	0.2	4.8 (0.54)
DCP/SC-MCC	21.8 (0.80)	0.5	13.2 (1.23)
DCP/Avicel PH 101	24.0 (0.56)	0.0	11.0 (0.46)
DCP (control)	18.2 (2.50)	0.0	>60

Values in parenthesis indicate standard deviation for two determinations. SC-MCC is microcrystalline cellulose from *Sorghum caudatum* and DCP is dicalcium phosphate dehydrate.

Dicalcium phosphate dehydrate is a free flowing material having no disintegrant properties[21]. The disintegration time values for compacts made from blend of DCP/cellulose powders (Table 4), shows the effects of the cellulose on the disintegration of tablets of this filler-binder. These values compared to over 60 min. disintegration time of tablets made without any cellulose revealed that these polymers have disintegrant properties in addition to the dry binding potential. This observation is in agreement with the reports that powdered microcrystalline cellulose has some disintegrant properties[22].

In conclusion, the cellulose product, SC-MCC, obtained from the stalk of *Sorghum caudatum* conformed to the official specifications in the British Pharmacopoeia (1993). The powder and tableting properties indicate that SC-MCC and Avicel PH101 are comparable, hence SC-MCC, is a potential dry binder and direct compression diluent in tableting.
